# Taxonomic Characterization, Antiviral Activity and Induction of Three New Kenalactams in *Nocardiopsis* sp. CG3

**DOI:** 10.1007/s00284-022-02954-x

**Published:** 2022-08-10

**Authors:** Omar Messaoudi, Eike Steinmann, Dimas Praditya, Mourad Bendahou, Joachim Wink

**Affiliations:** 1 Microbiology Laboratory Applied to Food Biomedical and Environmental (LAMAABE), Faculty of SNV-STU-Ex Imama Biomedical Complex, University of Abou Bekr Belkaid, PB 119, 13000 Tlemcen, Algeria; 2 Helmholtz Centre for Infection Research (HZI), Microbial Strain Collection, 38124 Brunswick, Germany; 3 Faculty of Science, Department of Biology, University of Amar Telidji, 03000 Laghouat, Algeria; 4 TWINCORE-Centre for Experimental and Clinical Infection Research (Institute of Experimental Virology), Hannover. Feodor-Lynen-Str. 7-9, 30625 Hannover, Germany; 5 Department of Molecular and Medical Virology, Ruhr-University Bochum, 44801 Bochum, Germany; 6 Research Center for Biotechnology, Indonesian Institute of Science, Jl. Raya Bogor KM 46, Cibinong, 16911 Indonesia

## Abstract

Exploration of secondary metabolites secreted by new *Actinobacteria* taxa isolated from unexplored areas, can increase the possibility to obtain new compounds which can be developed into new drugs for the treatment of serious diseases such as hepatitis C. In this context, one actinobacterial strain, CG3, has been selected based on the results of polyphasic characterization, which indicate that it represents a new putative species within the genus *Nocardiopsis*. Two fractions (F2 and F3), prepared from the culture of strain CG3 in soybean medium, exhibited a pronounced antiviral activity against the HCV strain Luc-Jc1. LC–HRESIMS analysis showed different bioactive compounds in both active fractions (F2 and F3), including five polyenic macrolactams (kenalactams A-E), three isoflavone metabolites, along with mitomycin C and one p-phenyl derivative. Furthermore, feeding with 1% of methionine, lysine or alanine as a unique nitrogen source, induced the production of three novel kenalactam derivatives.

**Supplementary Information:**

The online version contains supplementary material available at 10.1007/s00284-022-02954-x.

## Introduction

The challenge of the present medical research is the development of novel therapeutic agents [1]**,** which can facilitate to cure some serious diseases related to cancerous disease or infectious diseases caused by different bacteria or viruses such as Hepatitis C virus (HCV) [2] and SARS-CoV-2 [3]**.** HCV infects more than 1.5 million people worldwide every year, and it is considered as the major cause of liver cancer and cirrhosis [4]**.**

One of the most successful approaches to get new drugs is the exploration of the untapped habitats, such as saltpan, which provide the potential to isolate several novel microorganisms [5]**.** In fact, saltpans are ephemeral wetland with high concentration of salt and low amount of nutrients. They are the result of alternating periods of inundation and desiccation [6]**.** This extreme environment harbors many novel microorganisms, including *Actinobacteria* strains, able to synthesize different bioactive compounds. One of the widespread *Actinobacteria,* recovered, especially from diverse salty habitats, is the genus *Nocardiopsis* [7, 8]**.**

To survive under the extreme condition of saltpan, *Nocardiopsis* species developed distinct genetic and metabolic features and hold several biosynthetic gene clusters by which they acquire the ability to form an array of bioactive compounds [9, 10]**,** belonging to different chemical classes including: macrolides [11]**,** non ribosomal peptides [12]**,** macrolactams [13]**,** A-pyrones [14]**,** p-terphenyls [15]**,** diketopiperazine [16]**,** phenazine [17] and aziridine [18]**.** These compounds have been reported to display a potent biological activity [11, 12]**,** including: antibacterial [15, 18]**,** antifungal [19]**,** anticancer [20]**,** inflammatory [21]**,** antiparasite [22]**.** Therefore, species that belong to the *Nocardiopsis* genus, received a great attention**,** since they can provide a wide number of novel secondary metabolites which can help in creating new drugs in order to treat different serious disease [23]**.**

In this study, we report the taxonomic characterization of strain CG3, with polyphasic approach, and we conclude that this strain represents a new putative species in the genus *Nocardiopsis.* Afterward*,* we evaluate the antiviral activity of four fractions prepared from the culture of strain CG3 in soybean medium (SM) against the HCV strain Luc-Jc1. Finally, we determine the effect of different amino acids as sole nitrogen source on the biosynthesis of kenalactams (A-E) by CG3 strain, and we found that feeding with lysine or alanine as a starter, induces the biosynthesis of three novel kenalactams derivatives by the CG3 strain*.*

## Materials and Methods

### Isolation, Identification and Characterization of CG3 Strain

The strain CG3 (= DSM 106572^ T^ = NCCB 100649^ T^) used throughout this study, was selected from our collection of *Actinobacteria*, based on their taxonomic characterization performed with polyphasic approach.

The strain CG3 was isolated from the saltpan soil samples collected from Kenadsa region (Bechar, Algeria), using the starch casein agar medium. For molecular identification, the DNA extraction was performed using Invisorb Spin Plant Mini Kit, followed by PCR amplification of 16S rRNA *gene* region, using two primers, 27F and R1492 [24]**.** The PCR product was sequenced using five primers (F27, R518, F1100, R1100 and R1492) to obtain the complete 16S rRNA *gene* sequence, which can be compared with sequences present in the public sequence databases**.**

Phylogenetic tree was inferred from the sequences of 16S rRNA *gene* corresponding to the species belonging to the family of *Nocardiopsaceae*. The family of *Streptosporangiaceae* was used as outgroup. All used sequences were retrieved from GenBank. Phylogenetic analysis was conducted in RAxML [25]**,** using the maximum parsimony method [26]**.** The fast bootstrapping was used to generate the support values in the tree [27]**.**

The DNA–DNA hybridization was conducted between the strain CG3 and the closest species, according to the method of [28]**.** However, to calculate the G + C content (mole %) of the strain CG3, the DNA was extracted after cell disruption using a Constant Systems TS 0.75 KW instrument (IUL Instruments, Germany). The DNA was then purified on hydroxyapatit according to the procedure of Cashion et al. (1977) [29]. The obtaining DNA was hydrolyzed with P1 nuclease and the nucleotides were dephosphorylized with bovine alkaline phosphatase [30]**.** The resulting deoxyribonucleosides were analyzed by HPLC, using an Agilent 1260 Infinity II HPLC system equipped with a binary pump, a thermostatted automatic vial sampler and column compartment, and a HS diode array detector. Analytical HPLC conditions: the analytical column was an Infinity Lab Poroshell 120 EC-C18 (# 695,975-902 T; length 100 mm; diameter 4.6 mm; particle size 2.7 μm); solvent A: 20 mM ammonium acetate, solvent B: acetonitrile; gradient: 4% B, (0–1) min, (4–18.4) % B, (1–5) min; (18.4–40) % B, (5–6) min; pH 4.5; run time, 10 min. Flow rate, 1 ml/min. Temperature, 25 °C. Sample, 1 μl [29, 30]. The chromatograms were analyzed using the OpenLAB 2 software (Agilent, Santa Clara, CA, USA).

For chemotaxonomic characterization, the isomers of diaminopimelic acid were determined by the method of Hasegawa et al. (1983) [31], while, sugars in whole-cell hydrolyzates, were analyzed by TLC as described by Staneck et al. (1974) [32] and [30]. Polar lipids were analyzed according to the method of Minnikin et al. (1979) [33]. Cellular fatty acids, were analyzed by gas chromatography according to the method of Sasser (1990) [34], however, the respiratory quinones were identified according to the method of Tindall (1990a, 1990b) [35, 36].

Morphological, physiological, and biochemical characteristics of strain CG3 were investigated based on the protocol of Shirling & Gottlieb (1966) [35] and Williams et al. (1983) [38].

### Fermentation, extraction and the preparation of fractions from strain CG3

To prepare the seed culture, one piece (1 cm^3^) was cut from the well sporulated culture of CG3 strain grown on starch casein agar, and placed in an Erlenmeyer flask (250 mL) which contained 100 mL of soybean medium (SM): 2% mannitol, 2% soybean, 0.4% glucose, 3% NaCl, pH 7. After 7 days incubation at 37 °C on a rotary shaker (160 rpm), 80 ml of this culture was transferred to an Erlenmeyer flask (2 L of volume), contained 800 ml of SM to obtain the seed culture.

20 L of SM distributed in 25 Erlenmeyer flasks (2 L) each of which contain 800 ml SM, was inoculated with 10% (v/v) seed culture and incubated at 37 °C in a rotatory shaker with an agitation speed of 160 rpm. After 14 days incubation, the culture was centrifuged at 7000 rpm for 30 min, and the obtained biomass were then extracted three times with 1.5 L ethyl acetate, followed by centrifugation. The solvent was evaporated, the obtained organic extract was dissolved in 200 ml methanol and then subjected to n-heptane partition (V/V) in order to remove lipophilic components. After centrifugation, the methanol layer was evaporated to dryness and the crude extract (1.15 g) was redissolved in 200 mL methanol and fractionated using size exclusion chromatography (LH-20 column chromatography: 3 × 83 cm; flow rate: 3.8 mL/min), with Sephadex as the stationary phase, and the methanol as mobile phase. Four fractions were obtained (F1, F2, F3 and F4), which were evaporated and their antiviral activity was evaluated against HCV in human liver cells.

### Antiviral Activity Evaluation

To determine the antiviral activity of the fractions prepared from CG3 strain, the Human Hepatoma-derived cellular carcinoma cell line (HuH-7.5 FLuc) was used as the host cells for the propagation of the virus (HCV) in vitro**.** The cell line, HuH-7.5 FLuc, was derived via transduction of the gene encoding the enzyme Firefly luciferase (FLuc) [39]**.**

However, the Hepatitis C Virus strain used is Luc-Jc1, which is a chimeric HCV made by combining two genome segments derived from two HCV strains, such as J6CF (the origin of structural protein of the virus) and JFH1 (allows an efficient replication, in vitro, of the virus).

Moreover, the genome of strain Luc-Jc1 carries Renilla luciferase reporter (RLuc) [40]**.** Both genes, RLuc and FLuc, express stably the enzyme luciferase, which allows to easily quantify the viral replication (infectivity) and the cell viability, respectively [41]**.** In fact, in the presence of luciferin as substrates, the enzyme luciferase of both genes (RLuc and FLuc) produces light, detectable by a luminometer [42, 43]**.** This device quantifies the light produced in each well, and express the results as Relative Light Unit (RLU)/well.

The host cell line Huh7.5 Flu was inoculated in each well of a 12-well plate, containing Dulbecco’s modified minimum essential medium (DMEM, Life Technologies, Carlsbad, CA, USA, order number: 11965084.): 1 × minimum essential medium nonessential amino acids (MEM NEAA, Life Technologies), 2 mM glutamine, and 10% fetal bovine serum, in the presence of the four fractions (F1, F2, F3 and F4) prepared from the culture of strain CG3 in SM, at the concentration of 1 mg/mL. Penicillin and streptomycin are added at the concentraction of 100 IU/mL and 100 μg/mL, respectively, to prevent the cell cultures from bacterial contamination.Moreover, blasticidin (5 μg/mL) is added to the culture for selecting the cell line HuH-7.5 FLuc, which express the blasticidin resistance gene acquired via transfection.

The incubation was carried out under 5% CO_2_ atmosphere at 37 °C. After one hour, the host cell, Huh7.5 Flu, were inoculated with the RLuc Jc1 reporter virus in the presence of the four fractions (F1, F2, F3 and F4). Four hours later, the inoculum was removed and the adherent cells (monolayers) were washed three times with phosphate buffered saline (PBS), and then a fresh medium, without the inhibitor, was added [41].

Infected cells were lysed in 350 μl of lysis buffer (Trition-based), and then frozen at − 80 °C for 1 h following measurements of Renilla and Firefly luciferase activities on a Centro XS3 Microplate luminometer (Berthold Technologies, Bad Wildbad, Germany) as indicators of viral genome replication and cell viability, respectively [41].

### Effect of Different Amino Acids on the Biosynthesis of Kenalactams by CG3 Strain

Different L-amino acids, including: glutamic acid (Glu), lysine (Lys), valine (Val), arginine (Arg), alanine (Ala), phenylalanine (Phe), methionine (Met), glycine (Gly), histidine, (His), proline (Pro) and threonine (Thr), were selected to evaluate their effect on the biosynthesis of kenalactams (A-E) by strain CG3.

Feeding experiment was conducted in 50 mL Erlenmeyer flasks, containing 20 ml culture medium. The malt extract, which represent the nitrogen source in ISP2 medium, was substituted, separately, with 1% of each amino acid mentioned above. One Erlenmeyer flask containing ISP2 medium without malt extract, was used as negative control. The flasks were inoculated with 5% (v/v) seed culture, and incubated at 37 °C at 150 rpm on a rotary shaker. After 14 days incubation, 20 mL of each culture was extracted with the same volume of ethyl acetate in 50 ml Falcon tubes. The tubes were mixed for 30 min on a rotary shaker.

For separation of the organic phase from water phase the mixture was centrifuged at 9000 rpm for 10 min, the removed organic phase was evaporated to dryness and the crude extracts were redissolved in methanol.

An aliquot of 2 µL of each extract was subjected to Liquid Chromatography-High Resolution Electrospray Ionization Mass Spectrometry (LC-HRESIMS) analysis to detect the presence of kenalactams.

## Results and Discussion

### Taxonomic Characterization of Producing Strain

Recent trends in microbial metabolite research include the screening of secondary metabolites secreted by novel microbes. The main idea behind the use of new unknown microbial taxa, instead of known, is to increase the probability of discovering new metabolites, since new strains can enclose a new gene cluster for the biosynthesis of new molecules [44]**.**

In this context, and to pave the way for discovery of novel potent metabolite, one strain, CG3, isolated from untapped saltpan located in the Sahara of Algeria, has been selected because it has shown unique taxonomic characteristics compared to their closest neighbor species. A complete polyphasic characterization was undertaken to determine its taxonomic position.

**Fig. 1:**
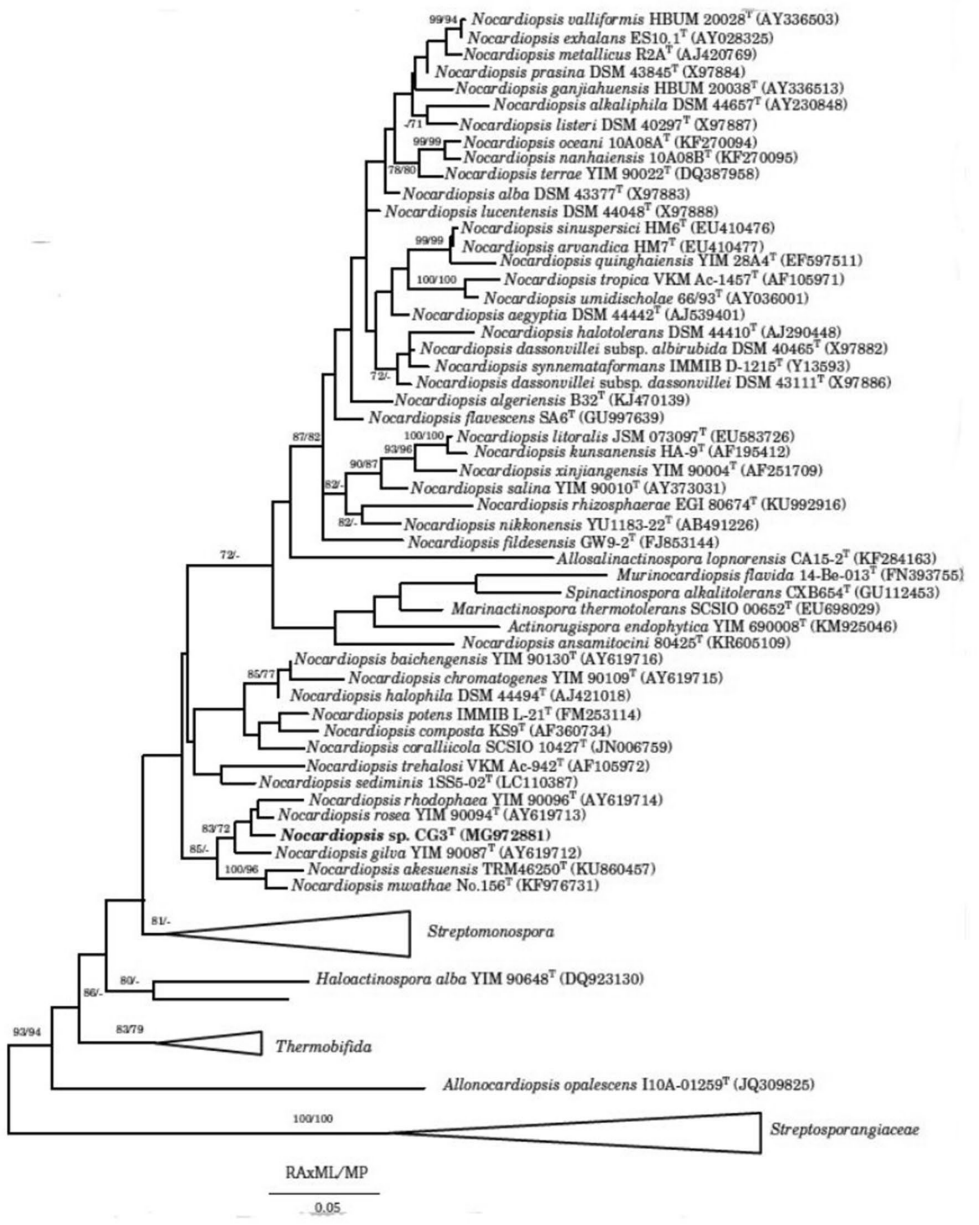
Maximum-parsimony phylogenetic tree [26], based on almost-complete 16S rRNA gene sequences (1502 nt) showing the position and phylogenetic relationship between CG3 and other related members of the new genus *Nocardiopsis*, and all type species of genera belong to the family of *Nocardiopsaceae*. The family of *Streptosporangiaceae* was used as outgroup. Numbers at the nodes are bootstrap values, expressed as a percentage of 1,000 resamplings (only values above 70% are shown)

Molecular identification of strain CG3, based on the 16S rRNA *gene* sequencing, showed the highest similarity to *Nocardiopsis rosea* YIM 90094^ T^ (99.2) %, followed by *N. gilva* YIM 90087^ T^ (98.5%) and *N. rhodophaea* YIM 90096^ T^ (98.2%). The phylogenetic tree, constructed based on the maximum parsimony method, showed that strain CG3 formed a distinguishable and stable sister branch within the clad formed by the type species *N. gilva* YIM 90087^ T^, *N. rhodophaea* YIM 90096^ T^ and *N. rosea* YIM 90094^ T^ (Fig. 1). Nouioui et al. [45], revealed that the clade formed by *N. gilva* YIM 90087^ T^, *N. rosea* YIM 90094^ T^ and *N. rhodophaea* YIM 90096^ T^, shows uncertain classification and is in need of revision.

*DNA–DNA* hybridization percentage between the strain CG3 and the closest species *Nocardiopsis rosea* YIM 90094^ T^ showed a relatedness values of 36%*,* which was significantly below the threshold for species delineation set to 70% [28]**.** Therefore, the strain CG3 represents a novel putative species within the genus of *Nocardiopsis*. However, to better distinguish the strain CG3 from its closest phylogenetic neighbors, further characterization, including chemotaxonomic, biochemical and phenotypic characterization, has been performed**.**

**Fig. 2:**
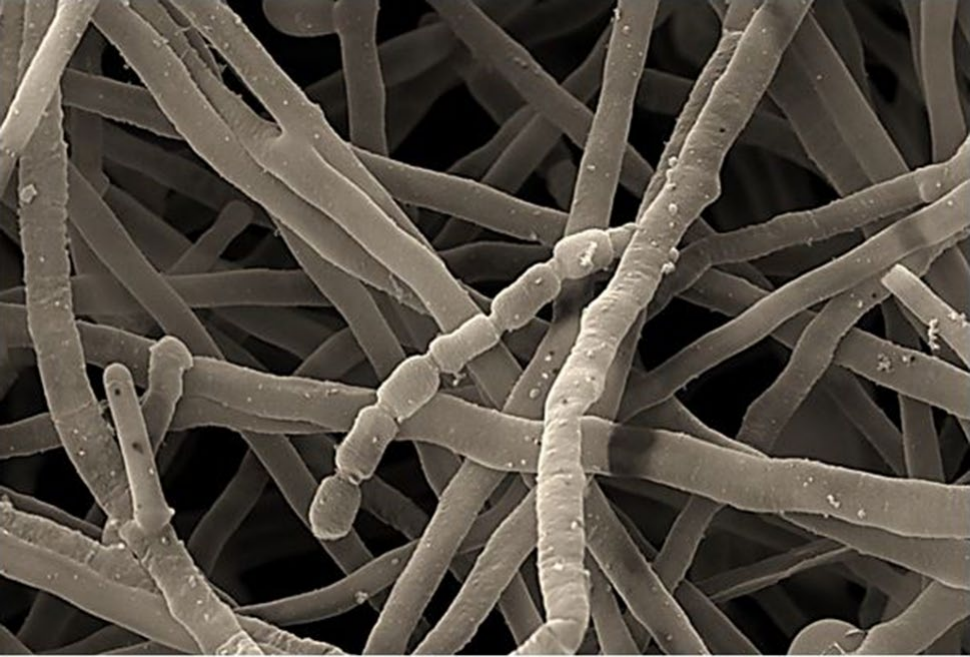
Scanning electron micrographs of strain CG3 showing straight short chains of smooth spores after growth on ISP3 agar supplemented by 3% Nacl for 18 days at 37 °C. Bars, 200 nm

The aerial mycelium of strain CG3 is well developed and bears short, smooth surface spore chains. In addition, unlike the species belonging to *Nocardiopsis* genus, the substrate mycelium formed by strain CG3 is stable (Fig. 2).

The cell–wall analysis of strain CG3, along with the three closely related species *N. rosea* YIM 90094^ T^, *N. gilva* YIM 90087^ T^ and *N. rhodophaea* YIM 90096^ T^, revealed the presence of meso-diaminopimelic acid, however, the glycine was absent. In addition, the ribose and galactose were the major diagnostic sugars (Supplementary Fig. S1) for the clade formed by strain CG3 and the three closest species, while, no diagnostic sugar characterizes the whole-cell hydrolyzates of species belong to *Nocardiopsis* genus [46]**.**

Furthermore, the major menaquinones found in strain CG3, together with the closest species were MK-11(H0, H2, H4, H6, H8) (Table 1), unlike the type species belong to *Nocardiopsis* genus which usually contained MK-9 (H4, H6) or MK- 10 (H2, H4, H6) [46]**.** Furthermore, the polar lipid pattern of strain CG3 was composed of phosphatidylcholine (PC), phosphatidylglycerol (PG), diphosphatidylglycerol (DPG), phosphatidylethanolamine, (PE), phosphatidylmethylethanolamine (PME), and phosphatidylinositolmannosides (PIM), phosphatidylinositol (PI) (Supplementary Fig. S2). Compared to the three closest species, *N. rosea* YIM 90094^ T^, *N. gilva* YIM 90087^ T^ and *N. rhodophaea* YIM 90096^ T^, two additional phospholipid (PL5 and PL10) were identified as the major polar lipids for strain CG3. However, this strain lacks one unknown phospholipid (PL4) and one major unidentified aminophospholipide (AL3) (Supplementary Fig. S2). Moreover, the major cellular fatty acids were iso-C 16: 0 (17,1%), iso-C 17:0 (13,5%), C 18:0 (11,4%), C 18:0 10- methyl (TBSA) (11.0%). The fatty acid profiles of strain CG3 were similar to their closely related species: *N. rosea* YIM 90,094 T, *N. gilva* YIM 90,087 T and *N. rhodophaea* YIM 90,096 T, although some quantitative and qualitative differences were noted (Table 1).

**Table 1:** Cellular fatty acid composition of strains CG3 and their most closely related neighbours in the genus *Nocardiopsis* [47]

Fatty acid	CG3	*N. rosea*	*N. gilva*	*N. rhodophaea*
Iso-C_14:0_	1.0	3.4	1.4	1.5
Iso-C_15:1_ G	–	–	1.1	–
Anteiso-C_15:1_ A	–	–	1.0	–
Iso-C_15:0_	2.7	3.0	2.4	4.7
Anteiso-C_15:0_	2.4	7.4	2.9	12.1
C_15:0_	-	1.0	0.3	0.6
Iso-C_16:1_ G	8.2	3.4	20.1	3.4
Iso-C_16:0_	17.1	28.4	6.9	15.7
C_16:1_ cis 9	-	2.3	3.6	1.7
C_16:0_	1.4	1.9	1.0	2.0
10-methl C_16:0_	3.5	2.1	8.1	1.6
Anteiso-C_17:1_ A	1.7	1.5	12.6	1.5
Anteiso-C_17:1_ C	–	–	–	–
Iso-C_17:0_	13.5	3.8	32	7.2
Anteiso-C_17:0_	8.7	10.8	3.4	15.6
C_17:1_ CIS 9	–	3.1	2.0	4.0
C_17:0_	2.2	1.7	0.8	2.8
10-methl C_17:0_	6.2	10.6	6.9	6.0
Iso-C_18:1_ G	–	–	1.0	–
Iso-C_18:0_	7.5	2.5	2.0	1.7
C_18:1_ CIS 9	0.7	2.2	2.7	5.0
C_18:0_	11.4	2.7	3.2	5.1
TBSA-C_18:0_ 10METHYL	11.0	6.5	12.9	8.0

The phylogenetic and chemotaxonomic study pointed out that the clade formed by *Nocardiopsis rosea* YIM 90094 T*, N. gilva* YIM 90087 T, *N. rhodophaea* YIM 90096 T and the strain CG3, is different compared to the other species belonging to *Nocardiopsis* genus. Therefore, this clade can be separated from the *Nocardiopsis* lineage and proposed as a new genus within the family of *Nocardiopsaceae*. However, to support this separation as a new genus in the family of *Nocardiopsaceae* further analysis like the whole genome sequencing of the new isolate CG3 and the multilocus sequence analysis (MLSA) have to be performed.

More phenotypic, physiological, biochemical and chemotaxonomical properties, which can distinguish the strain CG3 from its closest phylogenetic species are listed in Table 1 and 2, and Supplementary Table S1. From these data, it is evident that the strain CG3 represent a putative novel species within the genus *Nocardiopsis.* The GenBank accession numbers for the 16S rRNA gene sequences of the strain CG3 is MG972881.

**Table 2:** Characteristics differentiating the strain CG3 from closely related species [47]

Characteristic	*CG3*	*N. rosea*	*N. gilva*	*N. rhodophaea*
Vegetative hyphae	Stable	Fragmented	Fragmented	Fragmented
Temperature range for growth (°C)	25–50	20–60	10–40	20–60
Optimum temperature for growth (°C)	37–40	37–40	28–30	37–40
pH range/optimum	6–12/ Opt: 7.5	6–9/ Opt: 7.2	6–9/ Opt: 7.2	6–9/ Opt: 7.2
Nitrate reduction	+	+	+	–
Gelatin liquefaction	+	–	–	–
Urease	+	–	–	–
Carbon utilization D-Galactose D-Maltose L-Rhamnose D-Ribose D-Xylose L Arabinose D-Cellobiose D-Lactose D-Mannitol D-Raffinose D-Sorbitol	– + – + – – – – – – –	– + + + – + – + – – –	+ – – – + + + + + + +	– – – + – + – – – – –
Chemical characteristic Polar lipids	DPG, PG, PC PME, PE, PIM, PI, PL5, PL10, GL3, GL4	DPG, PG, PC PME, PE, PI, PL4, GL4 AL3, APL1	DPG, PG, PC PME, PE, PI, PL4, AL3, PL9, GL3	DPG, PG, PC PME, PE, PIM, PL4, GL4, AL3, PLx, APL1
Menaquinones	MH-11 (H0, H2, H4, H6, H8)	MK-11 (H0, H2, H4), MK-10 (H0, H2)	MK11- (H0, H2, H4, H6)	MK-11(H0, H2, H4, H6); MK-10 (H2)
Fatty acids	i-C16:1 G, i-C16:0, i-C17:0, ai-C17:0, C18:0, 10 Met C18:0	i-C16:0, ai-C17:0, 10-Met C17:0,	i-C16:1 G, 10-Met C18:0	i-C16:0, ai-C17:0, 10-Met C18:0
DNA G + C content (mol%)	69.6	67.9	68.1	69.0

### Antiviral Activity

**Fig. 3:**
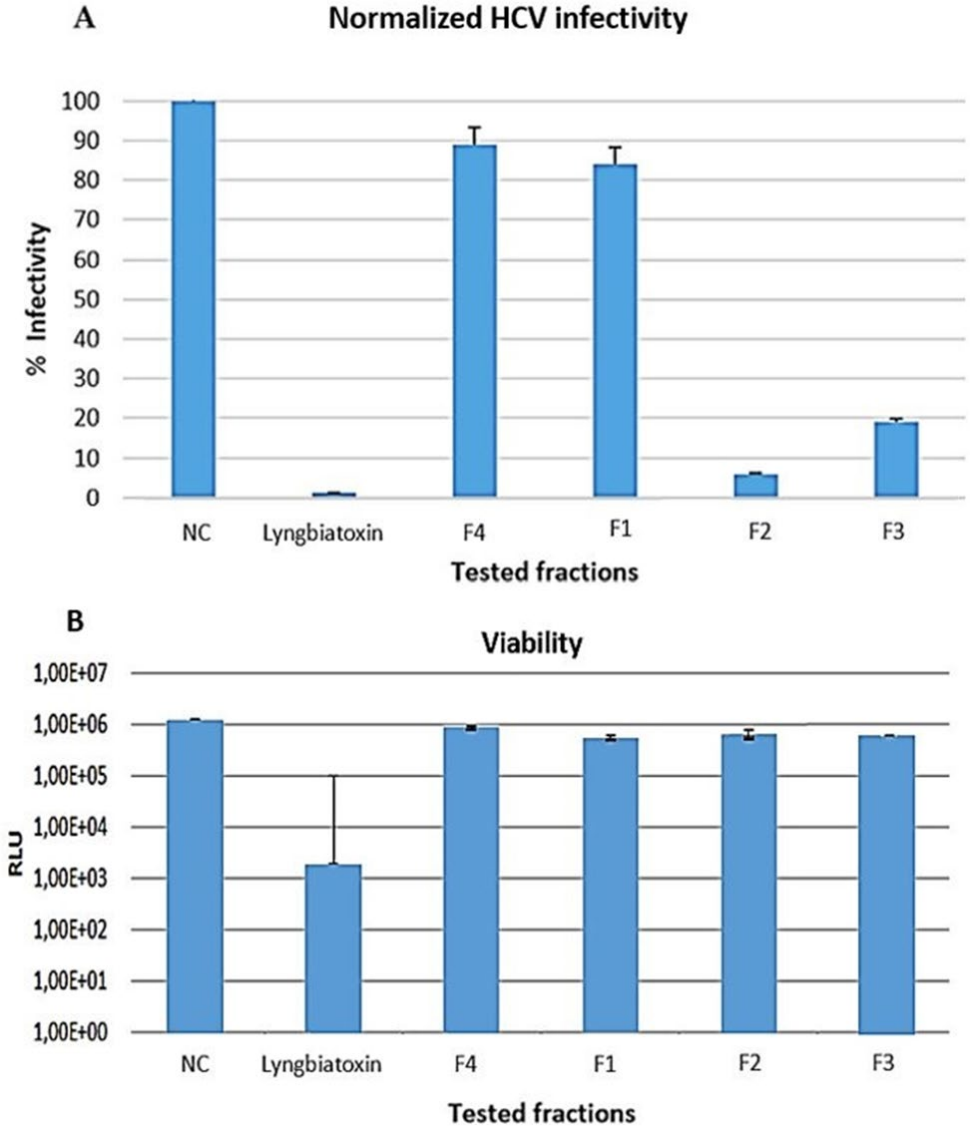
Antiviral activity against the HCV strain Luc-Jc1 of four fractions prepared from strain CG3. *F*1 fraction 1, *F*2: fraction 2, *F*3: fraction 3, *F*4: fraction 4, *NC* negative control, positive control: Lyngbiatoxin. The experiment was performed in duplicate and is presented with standard deviation. HuH-7.5 Fluc cells were infected with HCV strain Luc-Jc1 in the presence of fractions. The inoculum was removed after 4 h, and the monolayers formed were washed three times with PBS, afterward, a fresh medium without inhibitors was added. 3 days later, the infected cells were lysed and the infectivity **A** was estimated according to Renilla luciferase activity, however, the cell viability **B**, was determined according to the activity of Firefly luciferase

The antiviral activity of the four fractions (F1, F2, F3 and F4), prepared from the culture of strain CG3 in SM, were determined based on their ability to inhibit the HCV strain Luc-Jc1 to enter the host cell (HuH-7.5 Fluc) and multiply. In other words, this test aims to determine the capacity of each fraction to reduce the viral infectivity in cell culture. In parallel, the viability of the host cell (HuH-7.5 Fluc) towards the four fractions was also determined. The results are summarized in Fig. 3**.**

The viral infectivity was determined by the quantification of Renilla luciferase activity, which is maximal (100%) in negative control (well without fractions). The lower is the infectivity, the higher is the antiviral activity. Therefore, the two fractions (F2 and F3) reduce the infectivity of HCV strain Luc-Jc1 to 7% and 19%, respectively; consequently, both fractions exhibit a significant antiviral activity against Luc-Jc1 (Fig. 3 A). However, the fractions F4 and F1 were not active, since both fractions reduce the infectivity of the strain Luc-Jc1, to only 88% and 85% respectively (Fig. 3 A). In addition, the four fractions were not cytotoxic toward the host cell HuH-7.5 Fluc**,** compared to the positive control **(**Lyngbiatoxin) (Fig. 3 B).

From the results of Fig. 3, the putative new *Nocardiopsis* strain, CG3, might represent a good source of antiviral compounds. Moreover, little is known about the antiviral effect of the species belonging to *Nocardiopsis* genus, since until now, only one antiviral compound, K-252a, has been isolated from this genus. This molecule showed antiviral activity against the replication of vesicular stomatitis virus (VSV), in BHK-21 cells [48]**.** In addition, to the best of our knowledge, this is the first study aimed at to evaluate the antiviral activity of *Nocardiopsis* species against HCV.

The two active fractions (F2 and F3) prepared from the culture of strain CG3 in SM, were subjected to high-performance liquid chromatography coupled with high-resolution mass spectrometry (HPLC-ESI-HRMS), in order to identify the main bioactive metabolites in each fraction.

**Fig. 4:**
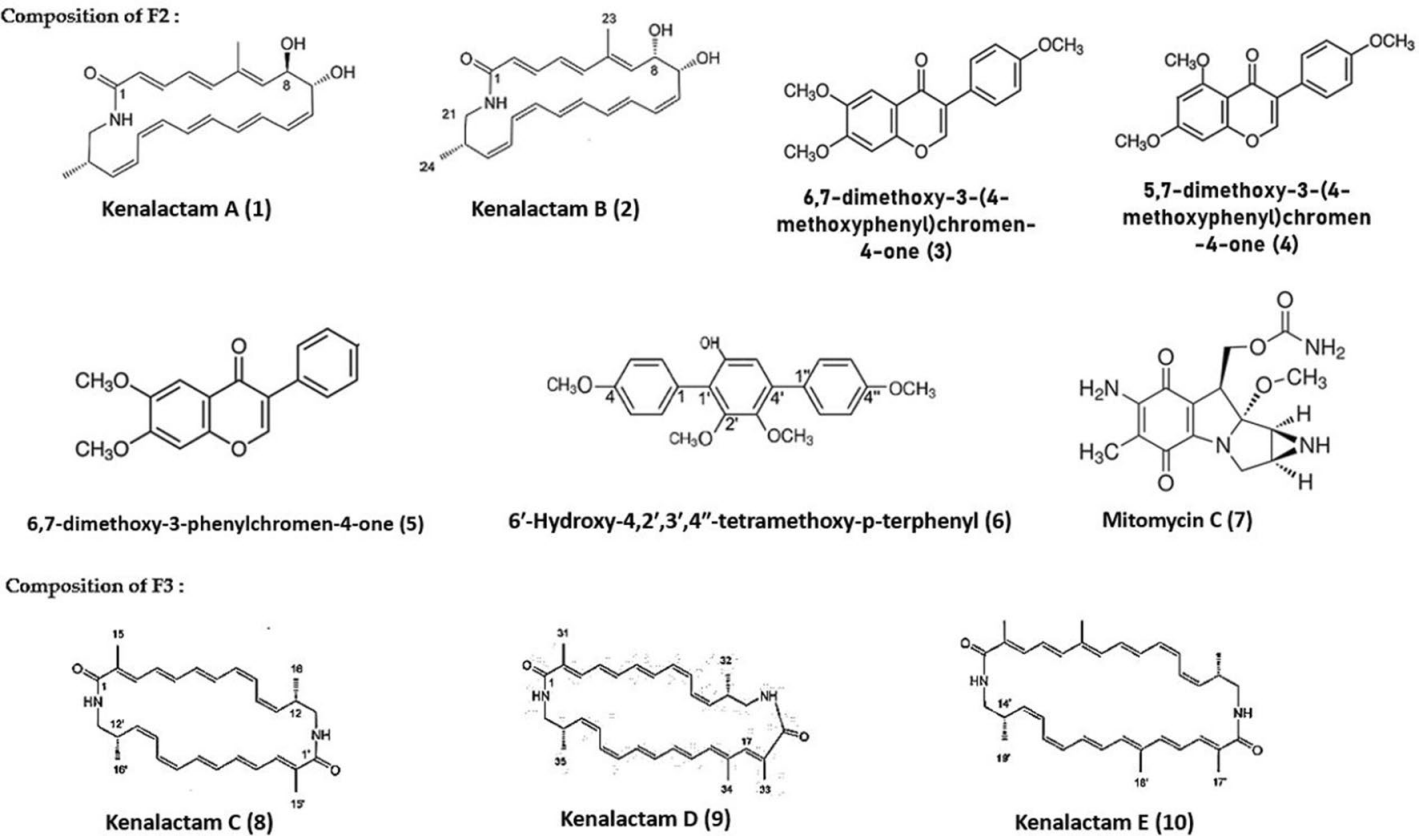
The main bioactive metabolites identified in F2 and F3

HPLC-ESI-HRMS analysis of F2 had led to the identification of two polyenic macrolactams, kenalactams A (1) and B (2), as well the three isoflavones compounds, 6,7-dimethoxy-3-(4-methoxyphenyl)chromen-4-one (3), 5,7-dimethoxy-3-(4-methoxyphenyl)chromen-4-one (4) and 6,7-dimethoxy-3-phenylchromen-4-one (5). In addition to the two known compounds 6'-hydroxy-4,2',3′,4''-tetramethoxy-p-terphenyl (6) and mitomycin C (7) (Fig. 4), unknown metabolites were detected (Supplementary Fig. S3)**.** Whereas, the major chemical compounds identified in the second active fraction, F3, were kenalactams C (8), D (9) and E (10) (Fig. 4), along with some unidentified compounds (Supplementary Fig. S4).

Furthermore, the antiviral activity of the pure five polyenic kenalactams (A-E) has been already evaluated during our previous study [13]**.** The results indicate that both kenalactams C and D reduce the infectivity of the HCV strain Luc-Jc1 to 60%. However, the kenalactams A and E reduce the infectivity of Luc-Jc1 to 80% and 75%, respectively, while, the antiviral activity of kenalactam B was not determined. In addition, the antiviral activity of F2 (Fig. 3) can be attributed to the combined action of the 1 and *2,* or to one of the other known compounds, besides kenalactams A and B, found in F2, which can act alone or in synergy. However, the antiviral activity of F3 (Fig. 3) can be linked to the synergistic effect between kenalactams C, D and E.

Little information exists in literature about the antiviral effect of macrolactams compounds. In fact, only fluvirucine which has been reported to exhibit an antiviral activity against the influenza A virus [49]**.** However, the compounds 3, 4 and 5 found in F2, as shown in Fig. 4, belong to isoflavone metabolites, known for their antiviral activity. For example, Arabyan et al., 2018 [50], indicate that the isoflavone, genistein shows activity against African swine fever virus (ASFV) with IC50 = 13 μM. Moreover, the class of terphenyl compounds, which include the p-terphenyl derivative 6 detected in F2 (Fig. 4), can exert an antiviral activity [51]. However, mitomycin C detected in F2 is not known to have antiviral activity.

Currently, the treatment of hepatitis C virus (HCV) infection has been transformed to the use of direct acting antivirals (DAAs), however, their cost remains a key barrier to access for many patients in a lot of countries [52]**.** Therefore, this study can provide the opportunities to develop a new antiviral drug, with a low cost, for the treatment of HCV disease, through, the purification, structure elucidation and the exploration of the mechanism of action of the antiviral metabolites, secreted by strain CG3, detected in F2 and F3.

### Effect of Different Amino Acids on the Biosynthesis of Kenalactams

The kenalactams (A-E) are a new family of polyene macrolactams secreted by the new species of *Nocardiopsis* sp. CG3. This kind of molecules are biosynthesized with the hybrid enzyme PKS-NRPS, which use different amino acids as a starter.

The amino acids are the origin of the secondary amine (-NH-) of the amide function within the macrolactam cycle [53]**.** The genes encoding macrolactam molecules, such as several secondary metabolites, are usually organized into cryptic genes and are regulated by an inducible operon [54]. Several methods have been developed in order to stimulate their expression, involving co-culture with other microorganisms, incubation under different culture condition (temperature, pH) and the use of chemical elicitor [55]**.**

**Fig. 5:**
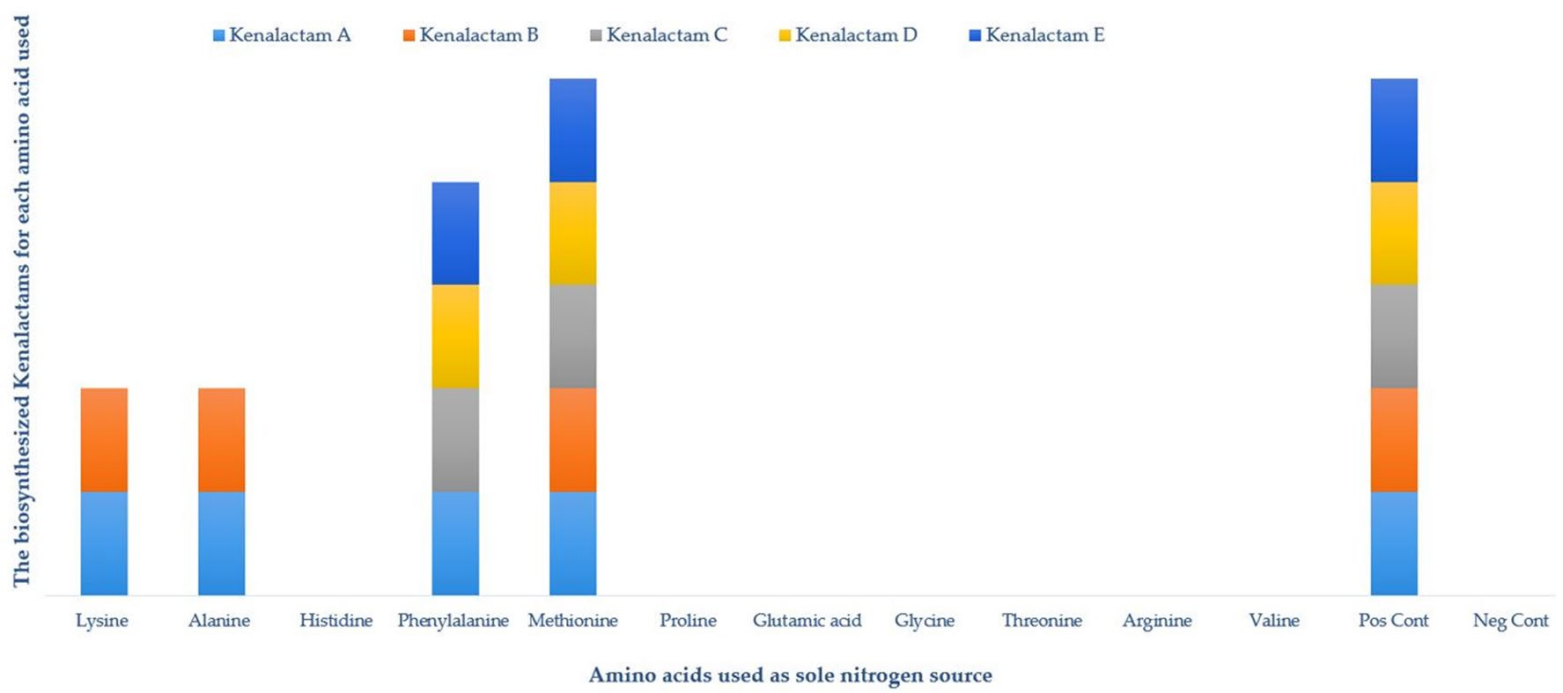
Effect of different amino acids used as sole nitrogen source in ISP2 medium, on the biosynthesis of kenalactams (**A-E**). Pos Cont: positive control (ISP2 with malt extract); Neg Cont: negative control (ISP2 without malt extract)

Feeding of strain CG3 with 11 different amino acids as sole nitrogen source, has been performed, to study their effect on the biosynthesis of kenalactams (A-E), as well as, to stimulate the cryptic genes for the biosynthesis of new kenalactams. The results are represented in Fig. 5.

As shown in Fig. 5, none of the five kenalactams (A-E) were detected in the crude extract prepared from negative control (ISP 2 without nitrogen source), however, all kenalactams (A-E) were present in the positive control (ISP 2 medium).

Only kenalactams A and B appeared when using the two amino acids, lysine and alanine, as sole nitrogen source in ISP 2 medium, however, when methionine was used as a starter, all kenalactams are produced by strain CG3 (Fig. 5). Furthermore, except kenalactam B, the kenalactams A, C, D and E are produced when phenylalanine was used as the unique nitrogen source (Fig. 5). Additionally, the strain CG3 cannot use the amino acids, proline, glutamic acid, glycine, threonine, arginine and valine, as a starter for the biosynthesis of kenalactams.

**Fig. 6:**
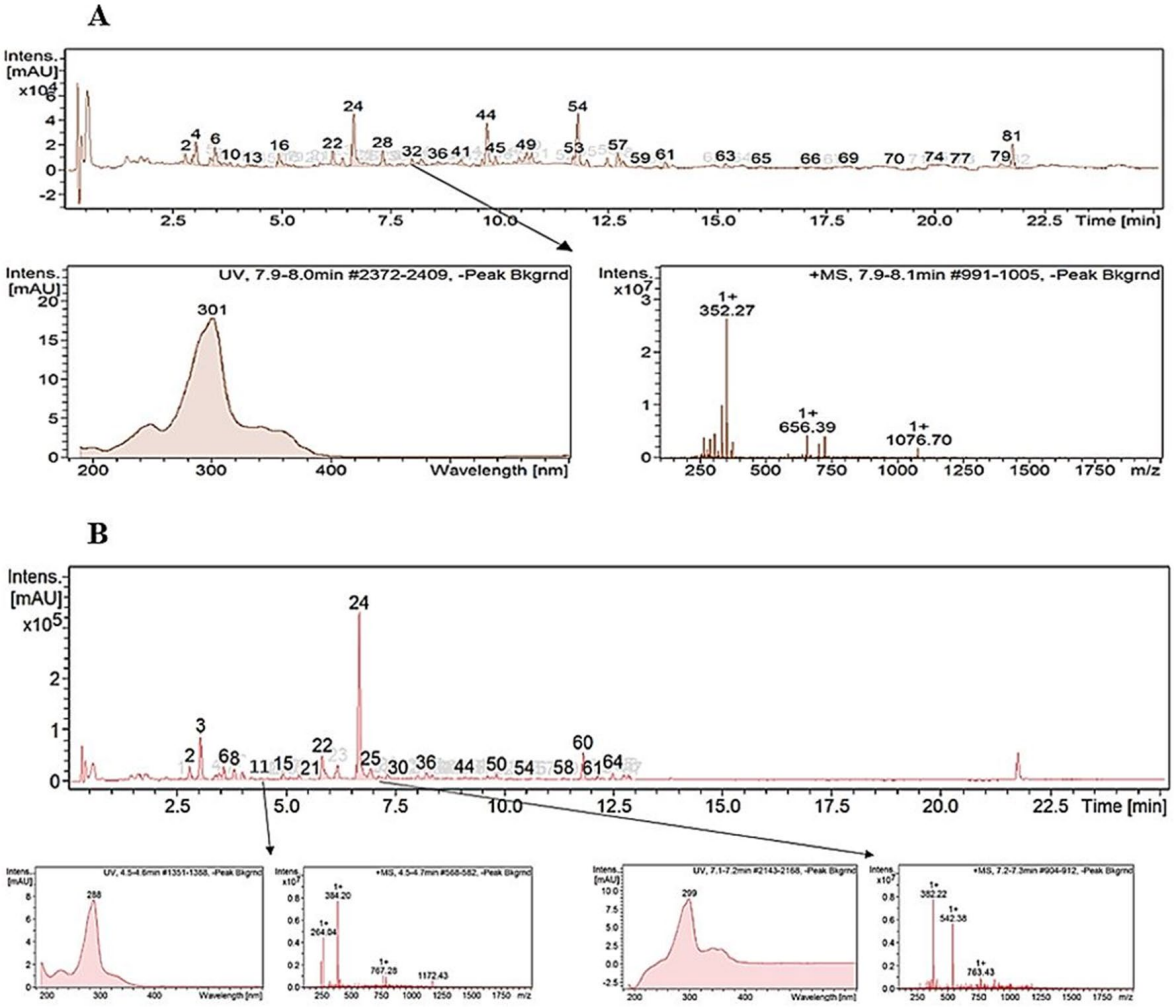
LC/HRESIMS analysis of crude extract prepared from the culture of strain CG3 in ISP2 medium. A: alanine was used as the unique nitrogen source in ISP2 medium, showing the induction of new peak of kenalactam (number 32). B: methionine was used as the unique nitrogen source in ISP2 medium, showing the induction of two new peaks of kenalactams (numbers 11 and 25)

Remarkably, the addition of 1% of alanine in ISP2 medium as a unique nitrogen source, induced the biosynthesis of a new metabolite (Rt = 8.00 min), characterized by the molecular ion cluster [M + H]^+^ at m/z 352.26 (32), which provides the molecular formula of C_22_H_25_NO_3_ (Fig. 6 A). However, two new peaks were detected at the retention time of 4.6 min (11) and 7.2 min (25), when methionine was used as a starter (Fig. 6 B). The molecular formula of both metabolites, 11 and 25, was determined as C_23_H_29_NO_4_ and C_24_H_31_NO_3_, on the basis of their molecular ion cluster of [M + H]^+^ at m/z 384.2 (11), and [M + H]^+^ at m/z 382.22 (25), respectively (Fig. 6 B). The three metabolites (11, 25 and 32), exhibited the same UV–visible absorbance spectrum maxima (248, 297 and 339 nm) as kenalactams A and B (Supplementary Fig. S5 and S6), therefore, they can be considered as new kenalactam derivatives.

The three peaks 11, 25 and 32 (Fig. 6), were completely absent in the negative control (ISP2 without nitrogen sources) (Supplementary Fig. S7), which confirm that the strain CG3 can produce the three metabolites (11, 25 and 32) only when the starters amino acids, alanine and methionine, are present in the culture medium.

**Fig. 7:**
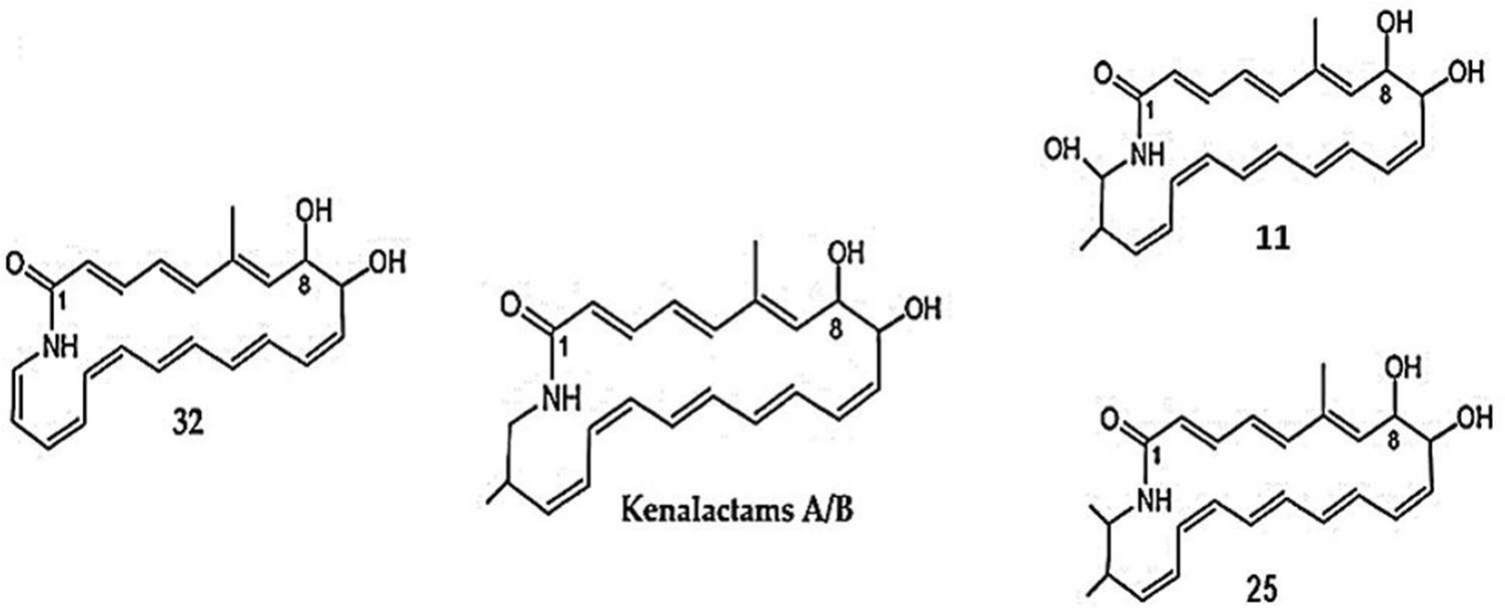
The proposed structure of the three new kenalactams derivatives (11, 25 and 32) inducted after feeding of strain CG3 with alanine and methionine as unique nitrogen source

By HPLC–UV-HRESIMS analysis, the structure of the three metabolites 11, 25 and 32 were deduced. In fact, the methyl group attached at C-20 in kenalactams (A or B) is absent in 32 (Fig. 7), whereas, an additional hydroxyl (OH) and methyl group (-CH_3_), can be linked to C-21 of kenalactams (A or B), which allowed to obtain the structure of 11 and 25, respectively (Fig. 7).

Purification and structure elucidation using NMR is necessary in order to confirm the structure of 11, 25 and 32.

## Conclusion

In summary, this study highlights for the first time the antiviral potential against HCV of species belonging to the genus *Nocardiopsis*. Despite the huge number of secondary metabolites providing by *Nocardiopsis* strains, this genus rests an untapped source for new antiviral drugs. In addition, feeding with lysine or alanine as starter, induces the biosynthesis of three new kenalactams derivatives by strain CG3. Nevertheless, more analysis is needed, regarding the identification of the antiviral compound (s) in F2 and F3 extracts, as well as the determination of their mechanism of action.

## Supplementary Information

Below is the link to the electronic supplementary material.

Supplementary file1 (RAR 2378 kb)
